# A Novel Pb-Resistant *Bacillus subtilis* Bacterium Isolate for Co-Biosorption of Hazardous Sb(III) and Pb(II): Thermodynamics and Application Strategy

**DOI:** 10.3390/ijerph15040702

**Published:** 2018-04-09

**Authors:** Yue Cai, Xiaoping Li, Dongying Liu, Changlin Xu, Yuwei Ai, Xuemeng Sun, Meng Zhang, Yu Gao, Yuchao Zhang, Tao Yang, Jingzhi Wang, Lijun Wang, Xiaoyun Li, Hongtao Yu

**Affiliations:** 1 Department of Environmental Science, School of Geography and Tourism, Shaanxi Normal University, Xi’an 710062, China; caiyue@snnu.edu.cn (Y.C.); liu_dongying@yeah.net (D.L.); jinse@snnu.edu.cn (C.X.); aiyuwei@snnu.edu.cn (Y.A.); sunxuemeng@snnu.edu.cn (X.S.); zhangmenghk@snnu.edu.cn (M.Z.); gaoyu1911@snnu.edu.cn (Y.G.); zyc@snnu.edu.cn (Y.Z.); yangfan2288@163.com (T.Y.); wangjz@snnu.edu.cn (J.W.); lijun_88@163.com (L.W.); lxy518@snnu.edu.cn (X.L.); 2 International Joint Research Centre of Shaanxi Province for Pollutant Exposure and Eco-Environmental Health, Xi’an 710062, China; hongtao.yu@morgan.edu; 3 School of Computer, Mathematical and Natural Sciences, Morgan State University, Baltimore, MD 21251, USA

**Keywords:** *Bacillus subtilis*, biosorption kinetics, Pb(II) and Sb(III), phytoremediation, thermodynamics

## Abstract

The present work is the first to study co-biosorption of Pb(II) and Sb(III) by a novel bacterium and its application strategy. The biosorption characteristics of Pb(II) and Sb(III) ions from aqueous solution using *B. subtilis* were investigated. Optimum pH, biomass dosage, contact time and temperature were determined to be 5.00, 6.00 mg/L, 45 min and 35 °C, respectively. Langmuir, Freundlich, Temkin and Dubinin-Radushkevich (D-R) models were applied to describe the biosorption isotherm of the metal ions by *B. subtilis*. Results showed that Langmuir model fitted the equilibrium data of Pb(II) better than others, while biosorption of Sb(III) obeyed the Freundlich model well. The biosorption capacity of *B. subtilis* biomass for Pb(II) and Sb(III) ions was found to be 17.34 ± 0.14 and 2.32 ± 0.30 mg/g, respectively. Kinetic data showed the biosorption process of Pb(II) and Sb(III) ions both followed the pseudo-second-order kinetic model, with R^2^ ranging from 0.974 to 0.999 for Pb(II) and from 0.967 to 0.979 for Sb(III). The calculated thermodynamic parameters, negative ∆*G* and positive ∆*H* and ∆*S* values, indicated the biosorption of Pb(II) and Sb(III) ions onto *B. subtilis* biomass in water was feasible, endothermic, and spontaneous. Bacterial bioleaching experiment revealed *B. subtilis* can increase the mobility of Pb(II) and Sb(III) in polluted soil when pH was close to 6 at low temperature. Consequently, *B. subtilis*, as a cheap and original bacterial material, could be a promising biomass to remove Pb or isolate Sb from industrial wastewater and to assist phytoremediation of Pb and Sb from weak acid or near neutral pH polluted soils at low temperature.

## 1. Introduction

Toxic heavy metal ions have a broad range of sources, especially in water resources [1]. Municipal wastes, mining and smelting of metalliferous ores, fertilizers, burning of fossil fuels, agriculture runoff and domestic effluent are the main sources of heavy metal contamination, which is difficult to remove and has detrimental effects on ecological systems and human health [2,3]. Among the various heavy metal ions presenting in wastewater, Pb(II) is one of the most prevalent. Sources of lead include tetraethyl lead-added gasoline, electrical storage batteries, mining, plating, lead smelting, photographic materials, explosive manufacturing, ammunitions, printing pigments, ceramic glass industries, etc. [4]. Lead has various harmful effects on human health [5]. Some of the known deleterious effects are with brain, kidney, reproductive system, nervous system, bone and heme synthesis [6]. It is, therefore, essential to remove Pb(II) from wastewater before disposal.

Antimony (Sb) is another well-known and hazardous toxic heavy metal with a particular ability to dissolve precious metals, such as gold. It was also used to purify gold from copper and silver until the 18th century. It is common to find compounds of antimony in silver, copper and lead containing ores. Anti-friction alloys, batteries, ammunitions like small arm and tracer bullets, type-metal and cable sheathing are main Sb-containing products since the use of Sb increases the hardness and the mechanical strength greatly. Sb also be used in brake linings, lead storage batteries, flame retardants, semiconductor components, as a catalyst in plastics, and an additive in glassware and ceramics. Consequently, it is introduced in the environment by anthropogenic pathways [7]. Sb(III) and Sb(V) ions, the main forms of Sb under environmental, biological and geochemical conditions, hydrolyze easily in aqueous solutions, with Sb(OH)_3_ and Sb(OH)_6_^−^ being the dominant chemical species in the aqueous environment [8]. Sb(III), however, is considered ten times more toxic than Sb(V) [9]. Due to the neutral character over a wide pH range of Sb(III), it is more strongly absorbed by its natural sorbents, making it more stable in the neutral environment [10]. Sb is classified as a toxic pollutant, potentially carcinogenic to humans and therefore as a pollutant of priority interest by entities such as the Environmental Protection Agency of the United States (USPEA) [11]. The USEPA’s maximum contaminant levels for Pb and Sb in drinking water is 15 μg/L and 6 μg/L, respectively [12,13]. Sb(III) is toxic for both the environment and human and it is a part of pollution resources the same as Pb(II). Sb and Pb, most of which was generated and then also consumed by the battery industry, and often coexist in industrial wastewaters or other Pb-Sb consumption source water systems. Therefore, attention should be paid to find out an efficient method to remove or isolate these two toxic heavy metal ions.

Ion exchange, reverse osmosis, membrane filtration, chemical precipitation, and evaporation are widely used methods for removing heavy metals from wastewater [14], but these technologies are either expensive for the treatment of secondary byproducts or ineffective when the concentration of metal ions is low. Sometimes, complex synthesis of exchangers is essential [15,16,17,18,19,20]. By contrast, biosorption is better because it is cheap and ecofriendly, especially when the biosorbents are original bacteria in soil. For biosorption, various biological materials have been used for removal, preconcentration and extraction of pollutants from aqueous solutions. For example, algae (e.g., *Sargassum nayans*, *Sargassum wightii*), fungi (e.g., *Aspergillus niger*, *Rhizopus arrhizus*, *Basidomycetes*, *Mucor Penicillium* spp.), bacteria (e.g., *Azotobacter*, *Ahthrobacter* spp., *Bacillus megaterium*, *Bacillus subtillis*, *Staphylococcus* spp., *Pseudomonas marginalis*) and yeast (e.g., *Saccharomyces cerevisae*) have already been used to remove heavy metal ions, due to their excellent recovery effect, eco-friendly, and economic features [21,22,23,24]. It has been found that bacteria are more efficient and cost-effective biosorption materials to remove toxic metals, especially for low concentrations of heavy metals in solution [25,26]. Bacteria, ubiquitous in soil and water, are a major group of unicellular living organisms belonging to the prokaryotes and are suitable as adsorbent materials due to their small size and large specific surface area. Most importantly chemical compounds presenting in a bacterial cell wall are capable of passively sequestering metals [27]. These chemical groups are comprised of carbonyl, hydroxyl, sulfhydryl, carboxyl, sulfonate, thioether, amine, amide, imine, phosphonate, imidazole, and phosphodiester groups. Factors such as accessibility of sites, affinity between sites and metals (i.e., binding strength), number of sites in the biosorbent materials and the chemical state of these sites (i.e., availability) all have influence on the importance of any given group for biosorption of a certain metal by a certain biomass.

Microbial populations living in metal polluted environments adapt to the toxic concentrations of heavy metals and become metal resistant [28]. In view of the interest in the use of microorganisms for the reclamation of contaminated sites, it is important to identify their response towards toxic heavy metals. In addition, studies of microbial populations in heavy metal polluted regions (especially in soil and water) are necessary since the microbes have adapted to survive in high heavy metal concentrations. The development of biosorbents isolated from polluted areas could be used to aid the advancement of alternative and cost-effective adsorbents. Recent studies have shown that some resistant bacterial microorganisms isolated from metal polluted sites are capable of absorbing Pb or Sb [29,30,31,32,33,34,35,36,37]. Although using these bacteria to remove Pb or Sb has been reported, it has been rarely studied using heavy metal resistant bacterial as biosorbent for biosorption and removal of Pb and Sb.

*Bacillus subtilis (B. subtilis)*, a rod-morphology bacterium, is a Gram positive aerobic spore, with a greater ability to bind metals than Gram-negative ones due to their different cell wall structures [38,39]. Teichoic acids and acids associated with the cell wall, whose phosphate groups are key components for the uptake of metals, are specific contents of Gram-positive cells. The main agents in the uptake of heavy metals are carboxyl groups, the sources of which are the teichoic acids associated with the peptidoglycan layers of the cell wall [40,41].

*B. subtilis* are inexpensive and easily available in the upper layers of the soil. Although there have been reports in the literature on the biosorption of Pb(II) by *B. subtilis* [32,42], there is no report on the biosorption of Pb(II) and Sb(III) together. Compared to the live biomass, dead bacterial biomass has a number of advantages, such as easy storage, the ability to treat large volumes of wastewater with low metal concentrations, short operation time, available with no harmful byproducts, and the absence of restrictions on enzymatic activities caused by metal adsorption [43]. In addition, the dead biomass does not require a continuous supply of nutrients, it is not affected by toxic wastes, and it can be regenerated and reused for many cycles. As a result, the usage of these dead microbial cells for adsorption is more advantageous for water treatment [44].

Therefore, the aim of this study is to explore and optimize the biosorption conditions of Pb(II) and Sb(III) in aqueous solution by a Pb-resistant bacterium, *B. subtilis*, in a batch system. The biosorption capacity obtained from the batch system is useful in providing fundamentals for industrial application of biosorption. The objective of the present work is to investigate: (1) thermodynamics and kinetics of biosorption of Sb(III) and Pb(II) ions onto *B. subtilis* biomass, (2) optimum biosorption parameters including pH, biomass dosage, contact time and temperature, (3) kinetic mechanism fitting to the Langmuir, Freundlich, Temkin and Dubinin-Radushkevich(D-R) models, (4) potential application strategy for using *B. subtilis* to remove Sb(III) and Pb(II) from water as well as its application prospectives for phytoremediation of Pb and Sb from polluted soils. 

## 2. Materials and Methods

### 2.1. Isolation and Identification of Bacillus subtilis

*B. subtilis* were isolated from soil samples collected from a 50-year old lead-contaminated alkaline soil. Soil (1.00 g) was added to 100.00 mL of sterile physiological salt solution in a flask and shaken at 80 rpm for 30 min. For isolation of lead-resistant bacteria, a series of 10-fold dilutions of soil suspensions were plated onto agar consisting of 3.00 g beef extract, 10.00 g peptone, 5.00 g NaCl and 15.00 g agar with a pH value of 7.00. Supplements, the lead acetate solutions, ranging from 50.00 to 1500.00 mg/L, were added to the nutrient broth. Then plates were incubated for 24 h at 37 °C. After the incubation periods, the Pb-resistant colonies were purified on the same media by streaking three to four times in fresh media. The purified isolated bacteria were centrifuged at 5500 rpm for 10 min and then separated from the growth medium and washed twice with physiologic serum. The selected isolates were cultured at 28 °C on trypticase soy broth agar for 48 ± 2 h and fatty acid methyl ester (FAME). MIDI protocol [45,46], gas chromatography with flame ionization detector (GC-FID) and the Microbial Identification Software (Newark, DE, USA) were used to analyze the isolates. As a known pathogen, the cultured *B. subtilis* were killed by drying at 70 °C for 24 h prior to further use. The live biomass has more hydrophobic entities on the surface of the cell wall and most of them were removed during the pretreatment. The treated biomass contained more available adsorptive sites on the surface, because of they lost their hydrophobic entities during treatment process. Thus, the dead biomass could have a better biosorption capacity.

### 2.2. Chemicals and Determination

Pb(II) solutions of different concentration were prepared from lead nitrate (Pb(NO_3_)_2_), Sb(III) solutions of different concentration were prepared from L-antimony potassium tartrate (LAPT, KSbC_4_H_4_O_7_), nitric acid and sodium hydroxide were used to adjust the pH. All the chemical reagents used in the present work were purchased from Sinopharm Chemical Reagent Co., Ltd. (Shanghai, China). These reagents were of analytical grade and used without further purification. An inductively coupled plasma atomic emission spectroscopy (ICP-AES) (Arcos, Spectro, Columbus, OH, USA) was used to determine the concentration of Pb(II) and Sb(III) ions. A Fourier transform infrared (FT-IR) spectrometer (Tensor27, Bruker, Billerica, MA, USA) was used to generate the different spectra. The pH measurement of solutions was carried out with a pH meter (PHSJ-4A, Shanghai Leici Company, Shanghai, China).

### 2.3. Batch Experiments

Batch experiments are affected by experimental parameters, which are important to evaluation of full biosorption potential of any biomaterial. This study focuses on: (i) solution pH, (ii) biomass dosage, (iii) temperature, (iv) contact time and period. Each batch experiment was performed in a 50-mL centrifuge tube with 20.00 mL Pb(NO_3_)_2_ solution (concentration of Pb(II) = 50.00 mg/L) and 20.00 mL LAPT solutions (concentration of Sb(III) = 50.00 mg/L) mixed in to make the concentration of Pb(II) and Sb(III) to be 25.00 mg/L. Total volume of the batch experiment was 40 mL.

#### 2.3.1. Effect of pH

This group experiments were carried out at 25 °C. The pH, ranging from 2.00 to 6.00, was adjusted with 1.00 mol/L HNO_3_. Then five centrifuge tubes were shaken for 12 h in an electrically thermostatic reciprocating shaker at 180 rpm. After shaking, tubes were centrifuged for 20 min at 3500 rpm. Finally, the supernatants were used to determine the concentration of Sb(III) and Pb(II). Each experiment was repeated three times to choose the optimum pH.

#### 2.3.2. Effect of Biomass Dosage

Seven groups of centrifuge tubes with mixed Pb(NO_3_)_2_ and LAPT solutions (under the optimum pH) were prepared to determine the optimum biomass dosage. For getting desired amount of biomass dosage, dry biomass powder was added accordingly to make 4.00, 8.00, 12.00, 16.00 and 20.00 mg/L respectively. The procedure was triplicate to obtain the optimum biomass dosage.

#### 2.3.3. Effect of Temperature

A series of centrifuge tubes with mixed Pb(NO_3_)_2_ and LAPT solutions (under the optimum pH and biomass dosage) were prepared to determine the optimum temperature. The temperature of the electrically thermostatic reciprocating shaker was changed to reach the reaction temperature at 15, 25, 35 and 45 °C, respectively. Solutions were kept for reaction at 180 rpm at different temperature for 12 h, after that solutions were centrifuged for 20 min at 3500 rpm. Then the supernatants were determined by ICP-AES. Experiments were repeated three times to obtain the optimum temperature.

#### 2.3.4. Effect of Contact Time

To achieve the optimum contact time, each mixed solution was under the optimum pH, biomass dosage and temperature. The series of centrifuge tubes were shaken in an electrically thermostatic reciprocating shaker at 180 rpm for 5, 8, 10, 15, 20, 25, 30, 45, 60, 90, 120 and 180 min respectively. Then centrifugation and determination were carried out as mentioned before.

### 2.4. Biosorption Isotherm Models

Isotherm adsorption equilibrium is often used to describe the equilibrium of the biosorption process by fitting the experimental points with models [47]. Four equilibrium models named Langmuir model, Freundlich model, Temkin model and Dubinin-Radushkevich (D-R) isotherm model were used to describe the biosorption isotherm of the metal ions onto *B. subtilis* biomass.

#### 2.4.1. Langmuir Isotherm Model

The Langmuir model is based on the hypothesis that uniform energies of adsorption onto adsorbent surfaces and the existence of monolayer coverage of the adsorbate at the homogeneous surface of the adsorbent where all sorption sites are identical and no transmigration of the sorbate. This model can be written as follows [48]:

(1) $$\frac{Ce}{q_{e}}=\frac{Ce}{q_{m}}+\frac{1}{K_{L}q_{m}}$$

where *q_e_* is the equilibrium metal ion concentration on the biosorbent (mg/g), *Ce* is equilibrium metal ion concentration in the solution (mg/L), *q_m_* is the monolayer biosoption capacity of the biosorbent (mg/g), and *K_L_* is the Langmuir biosoption constant (L/mg) related to the free energy of biosorption.

#### 2.4.2. Freundlich Isotherm Model

The Freundlich isotherm model suggests a monolayer sorption with a heterogeneous energetic distribution of active sites, accompanied by interactions between sorbed molecules. The Freundlich model [49] is:

(2) $$\log q_{e}=\log K_{F}+\frac{1}{n}\log Ce$$

where *K_F_* is a constant related to the biosoption capacity and 1/*n* is an empirical parameter related to the biosorption intensity, which varies with the heterogeneity of the biosorption materials.

#### 2.4.3. Temkin Isotherm Model

The Temkin isotherm is based on the assumption that the decline of the heat of sorption as a function of temperature is linear rather than logarithmic, as illustrated in the Freundlich equation [50]. Temkin isotherm has the form:

(3) $$q_{e}=\frac{RT}{b}\ln(aCe)$$

where *R* is the gas constant (0.0083 kJ/mol·K), *T* is absolute temperature (K), *b* is the Temkin constant in relation to heat of sorption (kJ/mol), *a* is the Temkin isotherm constant (L/g), *Ce* is equilibrium metal ion concentration in the solution (mg/L).

#### 2.4.4. Dubinin-Radushkevich (D-R) Isotherm Model

The D-R model fits the adsorption mechanism with a Gaussian energy distribution onto a heterogeneous surface [51]. The model has often successfully fitted high activities and the intermediate range of concentration data. The D-R isotherm model can be applied to evaluate the porosity characteristics of the sorbent and the apparent energy of adsorption. The liner form of the D-R isotherm equation is [52]:

(4) $$\ln q_{e}=\ln q_{m}-\beta \varepsilon^{2}$$

where *q_e_* is the amount of metal ion adsorbed on the per unit weight of biomass (mol/L), *q_m_* is the maximum biosorption capacity (mol/g), *β* is the activity coefficient related to biosorption mean free energy (mol^2^/J^2^) and *ε* is the Polanyi potential (*ε* = *RT* ln(1 + 1/Ce)).

### 2.5. Kinetic Models

One of the most important characteristics to describe the solute sorption rate is Kinetics of biosorption, which in turn controls the residence time of biomass sorption at the solid-solution interface. To clarify the biosorption kinetics of Pb(II) and Sb(III) on *B. subtilis* biomass two kinetics models, which are Lagergren’s pseudo-first-order and pseudo-second-order model were applied to the experimental data.

#### 2.5.1. Pseudo-First-Order Model

The pseudo-first-order model rate equation can be described as linearized form by [53]:

(5) $$\ln q_{e}(q_{e}-q_{t})=\ln q_{e}-k_{1}t$$

where *q_e_* is metal ions biosorbed at equilibrium (mg/g), *q_t_* (mg/g) is the amount of metal ions biosorbed at *t* (min) and *k*_1_ is the rate constant of the equation (min^−1^). The biosorption rate constant (*k*_1_) can be determined experimentally by plotting ln(*q_e_* − *q_t_*) vs. *t*.

#### 2.5.2. Pseudo-Second-Order Model

The Pseudo-second-order model can be given in the following form [54]:

(6) $$\frac{t}{q_{t}}=\frac{1}{k_{2}q_{e}^{2}}+\left(\frac{1}{q_{e}}\right)t$$

where *k*_2_ (g/mg min) is the rate constant of the second-order equation.

### 2.6. Microbial Bioleaching Experiment

Three experimental runs (numbered 1,2,3) were conducted in 50-mL centrifuge tubes. Runs 1 and 2 with 4.50 g soil added in were adjusted to an initial pH of 2.0 and 6.0, respectively. Quantitative biomass was added to make the concentration of *B. subtilis* to be 6.00 mg/L. A blank Run (3) without *B. subtilis* at each given pH was carried out as a control. Three reaction temperature series were set as 15, 25 and 35 °C which referred to the local weather temperature. Mixed solutions were shaken for 24 h at 160 rpm. After that, tubes were centrifuged for 35 min at 3500 rpm. Then supernatants were filtered with 0.22 µm filter membrane. Finally, filtered solutions were used to further determination. All treatments and control were done in triplicate.

## 3. Results

### 3.1. Optimum Conditions

#### 3.1.1. pH

**Figure 1:**
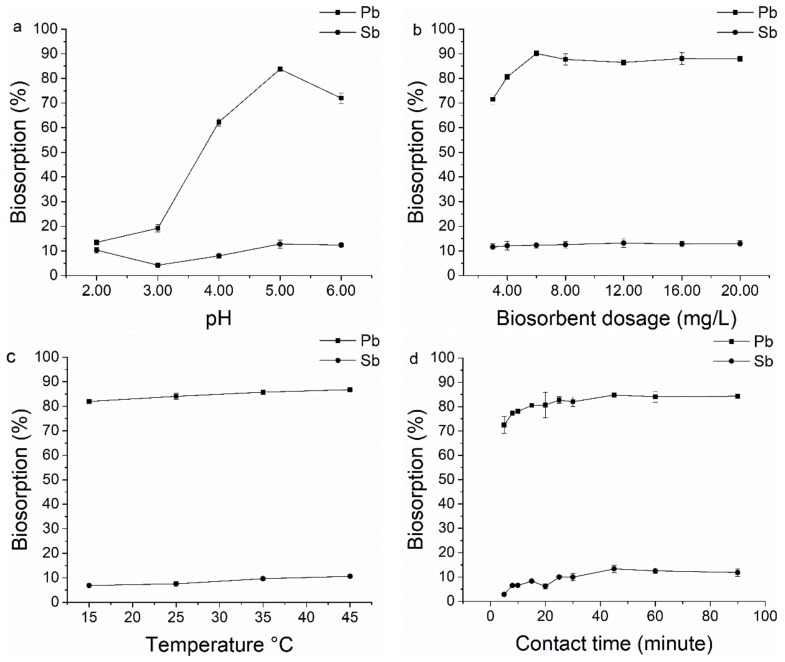
Effect of pH (**a**) metal concentration: 25.00 mg/L; temperature: 25 °C; biomass dosage (**b**) metal concentration: 25.00 mg/L; pH: 5.0, temperature: 25 °C; temperature (**c**) metal concentration: 25.00 mg/L; pH: 5.0, biomass dosage: 6.00 mg/L; and contact time (**d**) metal concentration: 25.00 mg/L; pH: 5.0, biomass dosage: 6.00 mg/L, temperature: 35 °C; on the biosorption of Pb(II) and Sb(III) ions on *Bacillus subtilis* biomass. Bars represented means ± SD (with three replicates).

Acidity of solution, affecting the competition of hydrogen ions with metal ions to active sites on the biosorbent surface, is one of the most important factors affecting biosorption of metal ions [55]. The effect of pH on the biosorption of Pb(II) and Sb(III) ions on *B. subtilis* biomass was studied by changing pH values ranging from 2.00 to 6.00 (Figure 1a). For Pb(II) ions, with the increase of pH from 2.00 to 5.00, the biosorption yield increased from 13.50% to 83.81% and the maximum biosorption yield was 83.8% when the pH was 5.00. At high pH values (pH > 5), the biosorption yields decreased. For Sb(III) ions, the biosorption yields were lower than Pb(II) ions, which were no more than 12.83%, no matter the pH values were low or high. Compared to Pb(II), Sb(III) had a lower biosorption (%), this observation was associated with Sb’s chemical speciation and behavior in water dependent of pH. Based on larger ionic radius of Sb and its lower charge density, Sb(III) ions hydrolyse easily in aqueous solution, thus making it difficult to maintain antimony ions stable in solution except in highly acidic media. It was found that Sb(III) was present as SbO^+^ (or Sb(OH)_2_^+^) under very acidic conditions and H_2_SbO_3_^−^ or Sb(OH)_4_^–^ was the main species present in mildly acidic, neutral, and alkaline conditions. As a result, all the following adsorption experiments were carried out at pH 5.00. The functional groups involved in metal uptake and metal chemistry could be used to explain pH dependency of biosorption efficiency [56]. At low pH, protons occupied most of the biosorption sites on the biomass surface and little Pb(II) (Pb^2+^) and Sb(III) (Sb(OH)_2_^+^) ions could be adsorbed because of electric repulsion with the protons on biomass. When the pH value increased, biosorbent surfaces were more negatively charged and the biosorption of the Pb ions with positive charges Pb^2+^ but Sb(III) became negatively charged H_2_SbO_3_^−^ or Sb(OH)_4_^–^ when the pH reached a maximum around 5.00. The decrease in biosorption at higher pH values (>5) was due to the formation of soluble negatively charged hydroxylated complexes of Pb and Sb ions, and their low absorption was attributed to the same charge between the bacterial surface and negative hydroxylated complexes of Pb (Pb(OH)_3_^−^ or Pb(OH)_4_^2−^) and Sb (H_2_SbO_3_^−^ or Sb(OH)_4_^–^). As a consequence, the retention would decrease again. In the present study, pH values higher than 6.00 were not studied, since it was expected that the majority of the wastewater contaminated with lead and antimony (from mining, for example) was acidic in nature.

#### 3.1.2. Biomass Dosage

The biomass efficiency for Pb(II) and Sb(III) ions as a function of biomass dosage was studied (Figure 1b). For Pb(II) ions, the percentage of the metal biosorption increase with the biomass loading up to 6.00 mg/L. The maximum biosorption, 90.81% of the metal ions, was attained at about biomass dosage 6.00 mg/L and it was slightly decreased at higher dosage 8.00, 10.00, 12.00 mg/L, then with the increase of biomass dosage the percentage of the metal biosorption increased but no more than 90.81%. For Sb(III) ions, the biosorption yields didn’t change obviously with the change of biomass dosage. This result can be explained that *B. subtilis* don’t have a large capacity to adsorb Sb(III) ions. At the beginning, the high concentration of metal ions compared to the accessible number of loaded surface-active groups in heat-inactive *B. subtilis* led to an active reaction. The decrease took place because of the partial aggregation, which occurred at higher biomass dosage giving rise in a decrease of active sites on the biomass [57]. Therefore, the amount of biomass was selected as 6.00 mg/L for further experiments.

#### 3.1.3. Temperature

Temperature of the reaction was an important factor during the process of biosorption [58], though various scenarios were demonstrated by different scholars. Effect of temperature on the biosorption of Pb(II) and Sb(III) ions on *B. subtilis* biomass was investigated (Figure 1c). The biosorption percentage increased from 84.11% to 89.73% for Pb(II) ions and from 5.88% to 13.87% for Sb(III) ions as temperature increased from 15 to 35 °C. This result indicated the endothermic nature of Pb(II) and Sb(III) ions biosorption on *B. subtilis*. Although biosorption yield had increased marginally when temperature was up to 45 °C, it was still very close to the biosorption capacity at 35 °C. It would consume more energy if the temperature were elevated to 45 °C. Therefore, in consideration of saving energy, an optimum temperature of 35 °C was selected for further biosorption experiments.

#### 3.1.4. Contact Time

Figure 1d shows the effect of contact time on the biosorption of Pb(II) and Sb(III) ions on *B. subtilis* biomass. It could be seen that the biosorption yields of Pb(II) and Sb(III) ions increased with rise in contact time up to 45 min, where the maximum of them were 84.85% and 13.36%, respectively. After that there was no considerable increase observed. Since a large number of vacant surface sites were available for the biosorption in the initial stage, the biosorption of metal ions was rapid and increased with time up to 45 min [38]. As a result, for further biosorption experiments the optimum contact time was selected as 45 min.

### 3.2. FT-IR Analysis

**Figure 2:**
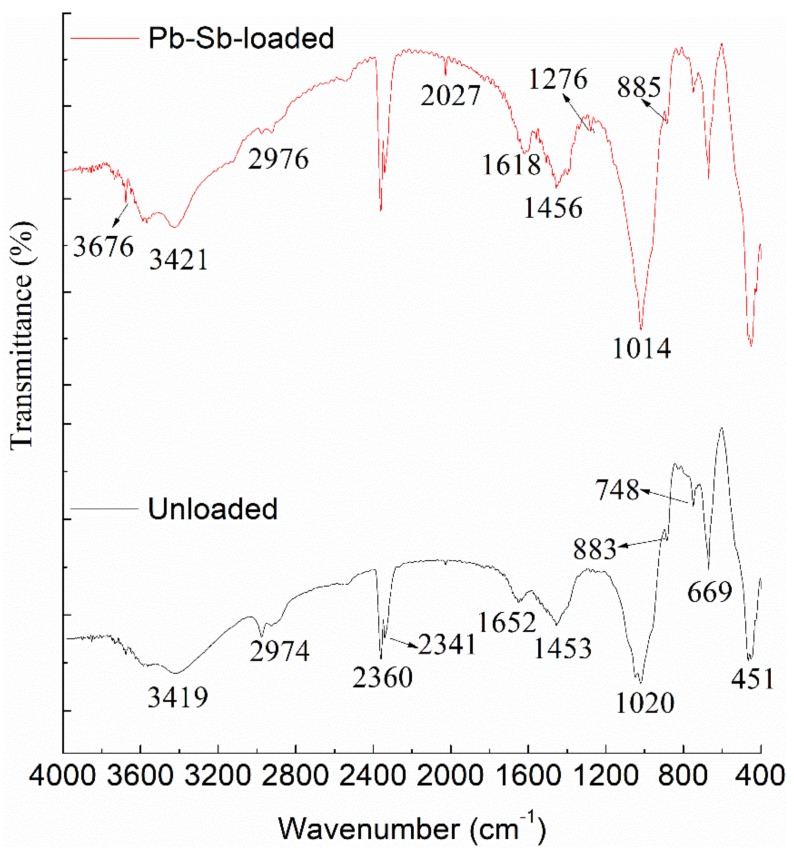
FT-IR spectrum of dried unloaded biomass and Pb-Sb-loaded biomass.

To determine which functional groups may contribute to the biosorption of Pb(II) and Sb(III) ions on the *B. subtilis* biomass surface, a FT-IR study was carried out. The spectra of dried unloaded biomass and Pb- and Sb-loaded biomass were recorded to obtain information on the nature of possible interactions between the functional groups of *B. subtilis* biomass and the metal ions (Figure 2). The spectrum of unloaded biomass exhibited a broad absorption band between 3750 and 3200 cm^−1^, which was due to bonded -OH or -NH groups. The peak at 2974 cm^−1^ was the indicator of -CH stretching vibration and the peak at 1652 cm^−1^ indicated a C=O stretching in carboxyl or amide I and amide II groups. The absorbance peak at 1456 cm^−1^ was attributed to N-H bending, -CH_2_ scissoring or -CH_3_ asymmetrical bending vibration and O-H deformation. The peak observed at 1020 cm^−1^ was assigned to C-O stretching of alcohols and carboxylic acids.

Conspicuous changes (appearance and disappearance of bands) were noted in the FTIR spectrum of the loaded biomass (Table 1). The stretching vibration at 3419 cm^−1^ shifted to 3421 cm^−1^ after the biosorption of Pb(II)-Sb(III), which indicated involvement of O-H groups in Pb^2+^ and Sb^3+^ binding. Changes in the OH adsorption peak indicated that the hydroxyl groups had been changed from multimeric to monopolymer or even a dissociative state [59], which showed that the degree of hydroxyl polymerization in the biomass surface was decreased by binding of Pb(II) and Sb(III), which offered more opportunity for Pb(II) and Sb(III) to be bound to the hydroxyl or amine groups. The peak of C-O group shifted to 1014 cm^−1^ after the biosorption of Pb(II)-Sb(III)-loaded. This result indicated that the free carboxyl groups changed into carboxylate ions, which occurred during the reaction of the metal ions and carboxyl groups of the biosorbent. Changes in the spectra were attributed to the interaction of Pb(II) and Sb(III) with the hydroxyl, carboxyl, sulfate and amino groups presenting on the surface of the biomass.

The FT-IR spectrum revealed that several types of functional groups presented in the structure of biomass and these groups can interact with metal ions from aqueous solutions during the sorption process. Therefore, metal ions could be bound by specific chemical interactions.

### 3.3. Biosorption Isotherm

**Figure 3:**
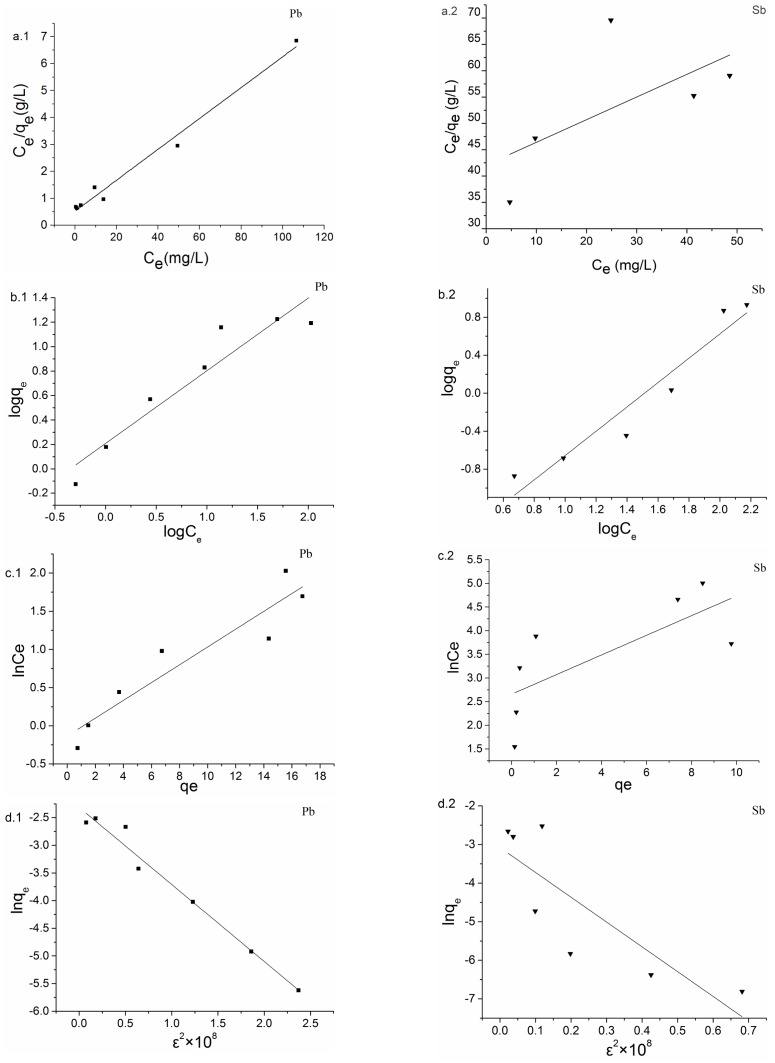
Langmuir (**a**), Freundlich (**b**), Temkin (**c**) and D-R (**d**) isotherm plots for the biosorption of Pb(II) and Sb(III) ions onto *B. subtilis* (pH: 5.00, biomass dosage: 6.00 mg/L, temperature: 35 °C; contact time 45 min).

It was essential to analyze the isotherm data to develop an equation which represented the results accurately and could be used for design purposes. In this study, four equilibrium models most commonly used in the literature were selected to describe the biosorption data, namely the Langmuir, Freundlich, Temkin and D-R isotherm equations. Table 2 shows the parameters and correlation coefficients of the different isotherm models. It could be concluded from the correlation coefficients that the Langmuir model fitted the biosorption of Pb(II) better than the other models, while a Freundlich model fitted the biosorption of Sb(III) well. Figure 3 shows the corresponding plots of the four models.

Figure 3a.1 indicates a linear relationship between the amount (mg) of Pb(II) ions sorbed per unit mass (g) of *B. subtilis* biomass against the concentration of Pb(II) ions remaining in solution (mg/L). The coefficient of determination (R^2^) was found to be 0.982, indicating the biosorption of the metal ions onto *B. subtilis* biomass fitted well the Langmuir model. In other words, the sorption of Pb(II) ions onto *B. subtilis* took place at the functional groups/binding sites on the surface of the biomass which is regarded as a monolayer biosorption process. The maximum biosorption capacity (*q_m_*) of *B. subtilis* biomass was found to be 17.43 ± 3.14 mg/g. Moreover, the *K_L_* value was found as 0.19 L/mg. The value of *K_L_* calculated from the Langmuir model was larger than that of previous study [60], which indicated *B. subtilis* biomass had a high biosorption affinity towards Pb(II) ions.

Figure 3b.2 shows the Freundlich isotherms obtained for the biosorption of Sb(III) ions onto *B. subtilis* biomass. The R^2^ was found to be 0.91, which was bigger than the R^2^ values of other models, indicating the Freundlich model was better than the other models at describing the relationship between the amount of Sb(III) sorbed by the biomass and its equilibrium concentration in the solution. The Freundlich isotherm model proposes a monolayer sorption and the energetic distribute on these active sites is heterogeneous. There are interactions between sorbed molecules [49]. Thus, it is suggested that biosorption of Sb(III) on *B. subtilis* biomass follow this kind of adsorption style.

### 3.4. Biosorption Kinetics

In order to clarify the biosorption kinetics of Pb(II) and Sb(III) ions on *B. subtilis* biomass, two kinetic models, Lagergren’s pseudo-first-order and pseudo-second-order model, were applied to the experimental data.

The plots of ln(*q_e_* − *q_t_*) vs. *t* for the pseudo-first-order model were not shown as figures because the coefficients of determination for this model at the studied temperature were low (R^2^ = 0.439–0.991 for the Pb(II) biosorption and R^2^ = −0.123–0.728 for the Sb(III) biosorption, as seen in Table 2). It can be concluded from the R^2^ values that biosorption mechanism of Pb(II) and Sb(III) ions on *B. subtilis* biomass did not follow the pseudo-first-order kinetic model.

**Figure 4:**
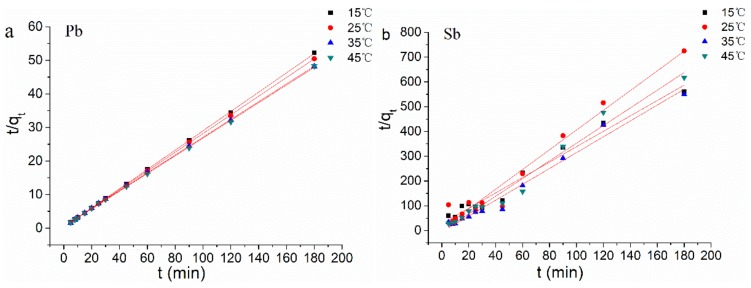
Pseudo-second-order kinetic plots at different temperatures: (**a**) for Pb(II) biosorption and (**b**) for Sb(III) biosorption.

Experimental data were tested by the pseudo-second-order kinetic model as well. This model was more likely to predict kinetic behavior of biosorption with chemical sorption being the rate-controlling step [61]. The linear plots of *t*/*q_t_* vs. *t* for the pseudo-second-order kinetic model for the biosorption of Pb(II) and Sb(III) ions on *B. subtilis* biomass at 15–45 °C were shown in Figure 4a and Figure 4b, respectively. Related parameters are given in Table 3. The R^2^ values were in range of 0.974–0.999 for Pb(II) biosorption and 0.967–0.979 for Sb(III) biosorption. These results indicated biosorption of Pb(II) and Sb(III) ions on *B. subtilis* biomass followed the pseudo-second-order kinetic model.

### 3.5. Biosorption Thermodynamics

In order to describe biosorption thermodynamic behavior of Pb(II) and Sb(III) ions on *B. subtilis* biomass, thermodynamic parameters, changes in free energy (∆*G*), enthalpy (∆*H*) and entropy (∆*S*) were calculated from following equation:

∆*G* = −*RT* ln*K_D_* (7)

where *R* is the universal constant (8.314 J/mol K), *T* is temperature (K) and *K_D_* (*q_e_*/*Ce*) is distribution coefficient.

The enthalpy (∆*H*) and entropy (∆*S*) parameters were estimated from the following equation:

(8) $$\ln K_{D}=\frac{△S}{R}-\frac{△H}{RT}$$

**Figure 5:**
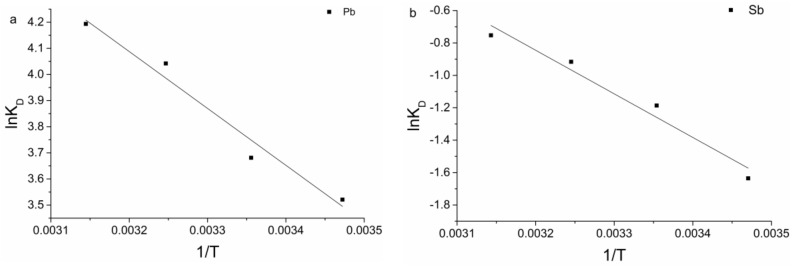
Plots of ln *K_D_* vs. 1/*T* for the estimate of thermodynamic parameters for biosorption on *B. subtilis* biomass (**a**) for Pb(II) and (**b**) for Sb(III).

Figure 5 shows the plot of ln*K_D_* vs. 1/*T*, according Equation (8), ∆*H* and ∆*S* can be calculated form the slope and intercept of the plot, respectively. Thermodynamic parameters under different temperatures are shown in Table 4. Gibbs free energy changes (∆*G*) were calculated to be −16.13, −17.09, −18.58 and −19.59 kJ/mol for Pb(II) biosorption and −4.23, −4.43, −5.38 and −5.63 kJ/mol for Sb(III) biosorption at 15, 25, 35 and 45 °C, respectively. The negative value of ∆*G* indicated spontaneous nature and thermodynamically feasible of the biosorption. The decrease in ∆*G* values with increase in temperature showed a decrease in feasibility of biosorption at higher temperature. ∆*H* parameters were found to be 18.10 and 22.37 kJ/mol for Pb(II) and Sb(III) biosorption, respectively. The positive ∆*H* indicated the endothermic nature of the biosorption process at 15–45 °C. The ∆*S* parameters were found to be 91.85 and 64.56 J/mol K for Pb(II) and Sb(III) biosorption, respectively. The positive ∆*S* value suggested an increase in the randomness at the solid/solution interface during the biosorption process.

## 4. Discussion

### 4.1. Strategy for Separation and Removal of Pb(II) and Sb(III) from Industrial Effluent and Wastewater

Since antimony and lead have commonly been used to produce batteries, Sb(III) and Pb(II) are usual ions in battery industrial wastewaters and effluents. Recently, the search for new treatment technologies for the removal of toxic metals from wastewater had directed attention to biosorption [62]. Biosorption could be considered as an alternative technology for industrial wastewater treatment [63], however, currently it is only practiced at lab scale despite several decades of development [64]. The mechanisms involved in biosorption or metal-microbe interactions should be further studied with great efforts by utilizing various techniques and combining some present ones together [65]. Using mathematical and dynamic models, in combination with molecular biotechnology tools, could be an available method for further elucidation. Various aspects shed light on the application of biosorption on an industrial scale including [66]:

- Physicochemical characteristics of ‘real’ wastewater on the basis of thermodynamics and reaction kinetics.
- Screening biosorbents with high metal-binding capacity and selectivity.
- Optimization of parameters.
- Combination of biosorption with physicochemical treatment technologies for ‘complete’ wastewater treatment and recovery/reuse of metals.

*Bacillus* bacteria have been widely considered by researchers for utilization in Pb(II) biosoption, but little information has been reported for Sb(III) biosorption and its removal from water (Table 5). In particular, when Pb(II) and Sb(III) co-exist in contaminated water, less attention had been paid to screening and use a capable bacterium to adsorb or separate Pb(II) and Sb(III) from water. The present study represents a small step towards such an approach by characterizing the biosorption process on the basis of thermodynamics and reaction kinetics and optimizing the process-affecting parameters. 

Our studies aimed at using a novel Pb-resistant *B. subtilis* bacterium isolate to co-adsorb hazardous Sb(III) and Pb(II) from water. This study also emphasized the application strategy for separating and removing Sb(III) and Pb(II) from Pb and Sb-contaminated industrial effluents according to the higher biosorption capacity of *B. subtilis* to Pb(II) than that of Sb(III) when pH = 5.00. It was noteworthy that metals could be removed from solution only when they were appropriately immobilized and the procedure of metal removal from aqueous solutions often led to effective concentration of the metal. That aspect of biosorption made the eventual recovery of this waste metal easier and economical, which will help the biosorption technology for large-scale application to separate or remove and recover Sb(III) and Pb(II) from battery manufacturing effluents.

### 4.2. Strategy for Possiblely Assisted Phytoremediation of Sb(III) and Pb(II) from Contaminated Soil 

**Figure 6:**
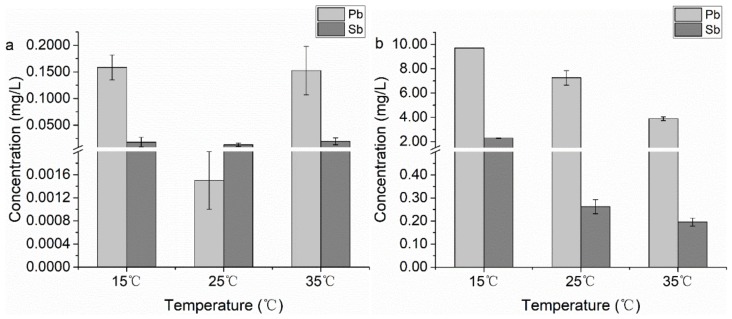
*B. subtilis* bioleaching of Sb(III) and Pb(II) pollution in the long-term contaminated real soil from battery manufacturing industries areas depending of different pH and temperature. (**a**) pH = 2.00, (**b**) pH = 6.00. Bars represent means ± SD (with three replicates).

*B. subtilis* are common soil microorganisms. The findings in the present study revealed that *B. subtilis* had different biosorption capacity depending on different pH conditions (Figure 1a). As a component of soil, *B. subtilis* with its functional groups could influence the Pb and Sb mobility in contaminated soils. Results from *B. subtilis* bioleaching of contaminated soil in Figure 6 display that *B. subtilis* had strong leaching capacity (removal) for Pb and Sb at pH = 6.00 (near the neutral) compared to pH = 2.00 (acidic conditions), which coincided with the results of our optimum pH experiments in water (Figure 1a). However, temperature (15–35 °C) had an obvious influence on the leaching capacity of Pb and Sb when the pH approached 6.00 (near the neutral point). On the contrary, when the pH changed to 2.00, the bioleaching varied, and results of bioleaching were not significant. This behavior could be explained by considering the charge variations of both adsorbent and adsorbate under different pH conditions. According to the speciation diagrams available in the literature [72], Sb(III) occurred in various speciation forms under different pH conditions, such as the form of Sb(OH)_2_^+^ at extreme acidic pH values, H_3_SbO_3_ or Sb(OH)_3_ in near neutral and slight alkaline conditions, and H_2_SbO_3_^−^ or Sb(OH)_4_^–^ at strong alkaline pHs. On the other hand, the charge of the adsorbent tends to be more positive at lower pH values. The bioleaching test showed that that *B. subtilis* had good leaching (removal) capacity for Pb(II) pollution in long-term contaminated soil at pH 6 and a low temperature (15 °C), which was the average temperature of the local autumn season. The studies elucidated the role of *B. subtilis* in bioleaching of Pb and Sb from near neutral pH soils at low temperature, which indicated that *B. subtilis* could increase the mobility of Pb and Sb in polluted soil at low temperature. *B. subtilis* conventionally exists in soil, has good biosorption capacity for Pb(II) and Sb(III) in water and also good bioleaching capacity for them in soil under weakly acidic or near neutral pH conditions. Therefore, the application of microorganism-assisted remediation based on *B. subtilis* is a promising strategy for improving the mobility of Pb and Sb. *B. subtilis* biomass could be used as a phytoremediation-assisted material for removal of Pb and Sb from weakly acid or near neutral pH polluted soils at low temperature.

## 5. Conclusions

This study is the first to investigate the co-biosorption from aqueous solution of Pb(II) and Sb(III) ions onto a novel Pb-resistant bacterial biomass, *B. subtilis*, and discuss an application strategy based on isotherm thermodynamics and bioleaching. Optimum thermodynamic parameters of biosorption, including solution pH, biomass dosage, temperature and contact time were selected. It was found that the biosorption capacity of Pb(II) was 17.34 ± 0.14 mg/g under the optimum conditions of pH = 5.00, biomass dosage 6.00 g/L, contact time 45 min, temperature 35 °C, however, the biosorption capacity of Sb(III) under the same conditions was 2.32 ± 0.30 mg/g. Tests of the experimental data with respect to kinetic models showed that the biosorption of Pb(II) and Sb(III) ions onto *B. subtilis* biomass followed a pseudo-second-order kinetic model. Negative values of ∆*G* and positive ∆*H* and ∆*S* indicated the feasibility, endothermic and spontaneous nature of the biosorption at 15–45 °C. It was also noteworthy that when the pH was close to 6.00, the *B. subtilis* bioleaching capacity to Sb(III) and Pb(II) in alkaline soil was obviously improved, which indicated that *B. subtilis* could potentially enhance the mobility of Sb(III) and Pb(II) in soils. Although *B. subtilis* had lower biosorption capacities for Sb(III) and Pb(II) at 15–45 °C than other biomasses, the significant importance of this study could provide application strategies of *B. subtilis* not only to remove Pb(II) or isolate Sb(III) ions from industrial effluents, especially battery manufacturing wastewaters where Pb and Sb coexist, but also to prospectively assist the phytoremediation for Pb and Sb in contaminated alkaline soils.

## Figures and Tables

**Table 1:** Assignment of FT-IR spectroscopy of dried unloaded and Pb-Sb-loaded *B. subtilis* biomass.

Wavelength (cm^−1^)	Unloaded Cell (cm^−1^)	Pb-Sb-Loaded Cell (cm^−1^)	Assignment
3750–3200	3419	3676, 3421	bonded -OH or -NH groups
3000–2850	2974	2976	-CH stretching vibration.
1680–1550	1652	1618	C=O stretching in carboxyl or amide I and amide II groups
1550–1375	1453	1456	N-H bending, -CH_2_ scissoring or -CH_3_ asymmetrical bending vibration and O-H deformation
1375–1300	-	1276	C-O stretching of -COOH
1200–1000	1020	1014	C-O stretching of alcohols and carboxylic acids
1000–800	883	885	S=O stretching

**Table 2:** Parameters of different isotherm models for biosorption of Pb(II) and Sb(III) by *B. subtilis* biomass.

Isotherm Model	Parameter	Pb(II)	Sb(III)
Langmuir	*q*_max_ (mg/g)	17.43	2.32
	*K_L_* (L/mg)	0.19	0.010
	R^2^	0.98	0.20
Freundlich	*k_F_* (L/g)^1/*n*^	1.62	19.23
	*n*	1.68	0.78
	R^2^	0.89	0.91
Temkin	a	1.42	0.070
	b	293.76	522.47
	R^2^	0.87	0.45
D-R	*q*_max_ (mg/g)	0.099	0.046
	*β*	1.39	6.43
	R^2^	0.97	0.64

**Table 3:** Kinetic parameters obtained from pseudo-first-order and pseudo-second-order model for the biosorption of Pb(II) and Sb(III) ions on *B. subtilis* biomass at different temperatures.

T (°C)		*q_e, exp_*	Pseudo First Order Equation			Pseudo Second Order Equation		
		(mg/g)	*k*_1_ (min^−1^)	*q_e_* (mg/g)	R^2^	*k*_2_ (g/mg min)	*q_e_* (mg/g)	R^2^
Pb(II)	15	3.489	0.012	0.210	0.439	0.405	3.474	0.999
	25	3.577	0.020	0.311	0.842	0.251	3.589	0.999
	35	3.737	0.030	0.695	0.957	0.124	3.772	0.999
	45	3.791	0.040	0.879	0.991	0.150	3.795	0.974
Sb(III)	15	0.322	0.0073	0.199	0.728	0.345	0.322	0.974
	25	0.248	0.0047	0.118	0.251	2.027	0.251	0.967
	35	0.328	−0.0004	0.062	−0.123	−1.947	0.313	0.979
	45	0.292	0.0014	0.127	−0.089	−3.340	0.281	0.976

**Table 4:** Thermodynamic parameters under different temperature.

Parameter	∆*G* (KJ/mol)		∆*H* (KJ/mol)		∆*S* KJ/(mol·K)	
Temperature (°C)	Pb	Sb	Pb	Sb	Pb	Sb
15	−16.13	−4.23	18.10	22.37	91.85	64.56
25	−17.09	−4.43				
35	−18.58	−5.38				
45	−19.59	−5.63				

**Table 5:** Lead and antimony adsorption capacity with different types of biological materials.

Pb(II)				Sb(III)			
Biosorbent	*q_m_* (mg/g)	Biosorption Mechanism	Reference	Biosorbent	*q_m_* (mg/g)	Biosorption Mechanism	Reference
*Bacillus subtilis*	17.34	L	this study	*Bacillus subtilis*	2.32	F	this study
*Bacillus cereus*	23.3	L	[38]	*Brown Algae*	5.5	L	[7]
*Bacillus pumilus*	29.6	L	[38]	*Cyanobacterium Synechocystis* sp.	4.68	L	[67]
*Bacillus cereus*	72.0	R-P	[68]	*Microcystis*	4.88	L	[69]
*Bacillus* sp. *(ATS-1)*	92.3	L	[70]				
*Bacillus cereus*	36.7	L	[71]				
*Bacillus subtilis*	57.0	Not mention	[32]				

Note: L: Langmuir model; F: Freundlich model; R-P: Redlich-Peterson Model.

## References

[1] Mahmoud M.E., Nabil G.M., Mahmoud S.M.E. (2015). High Performance Nano-Zirconium Silicate Adsorbent for Efficient Removal of Copper(II), Cadmium(II) and Lead(II). J. Environ. Chem. Eng..

[2] Wu S.C., Peng X.L., Cheung K.C., Liu S.L., Wong M.H. (2009). Adsorption kinetics of Pb and Cd by two plant growth promoting rhizobacteria. Bioresour. Technol..

[3] Gupta V.K., Rastogi A. (2008). Biosorption of lead(II) from aqueous solutions by non-living algal biomass *Oedogonium* sp. and *Nostoc* sp.—A comparative study. Colloids Surf. B Biointerfaces.

[4] Singh V., Tiwari S., Sharma A.K., Sanghi R. (2007). Removal of lead from aqueous solutions using *Cassia grandis* seed gum-graft-poly(methylmethacrylate). J. Colloid Interface Sci..

[5] Velmurugan N., Hwang G., Sathishkumar M., Choi T.K., Lee K.J., Oh B.T., Lee Y.S. (2010). Isolation, identification, Pb(II) biosorption isotherms and kinetics of a lead adsorbing *Penicillium* sp. MRF-1 from South Korean mine soil. J. Environ. Sci..

[6] Hasan S.H., Srivastava P., Talat M. (2009). Biosorption of Pb(II) from water using biomass of Aeromonas hydrophila: Central composite design for optimization of process variables. J. Hazard. Mater..

[7] Ungureanu G., Santos S., Boaventura R., Botelho C. (2015). Biosorption of antimony by brown algae *S. muticum* and *A. nodosum*. Environ. Eng. Manag. J..

[8] Okkenhaug G., Zhu Y.G., Luo L., Lei M., Li X., Mulder J. (2011). Distribution, speciation and availability of antimony (Sb) in soils and terrestrial plants from an active Sb mining area. Environ. Pollut..

[9] Sigel H., Sigel A., Sigel H. (1994). Handbook on Metals in Clinical and Analytical Chemistry.

[10] Johnson C.A., Moench H., Wersin P., Kugler P., Wenger C. (2005). Solubility of antimony and other elements in samples taken from shooting ranges. J. Environ. Qual..

[11] USEPA (1979). Water Related Fate of the 129 Priority Pollutants.

[12] USEPA (1999). National Primary Drinking Water Standards.

[13] USEPA (2013). Lead in Drinking Water.

[14] Pan X., Wang J., Zhang D. (2005). Biosorption of Pb(II) by Pleurotus Ostreatus Immobilized in Calcium Alginate Gel. Process Biochem..

[15] Naushad M., ALOthman Z.A., Awual M.R., Alam M.M., Eldesoky G.E. (2015). Adsorption kinetics, isotherms, and thermodynamic studies for the adsorption of Pb^2+^ and Hg^2+^ metal ions from aqueous medium using Ti(IV) iodovanadate cation exchanger. Ionics.

[16] Ghasemi M., Naushad M., Ghasemi N., Khosravi-Fard Y. (2014). Adsorption of Pb(II) from aqueous solution using new adsorbents prepared from agricultural waste: Adsorption isotherm and kinetic studies. J. Ind. Eng. Chem..

[17] Al-Othman Z.A., Naushad M., Nilchi A. (2011). Development, Characterization and Ion Exchange Thermodynamics for a New Crystalline Composite Cation Exchange Material: Application for the Removal of Pb^2+^ Ion from a Standard Sample (Rompin Hematite). J. Inorg. Organomet. Polym. Mater..

[18] Bushra R., Naushad M., Adnan R., ALOthman Z.A., Rafatullah M. (2015). Polyaniline supported nanocomposite cation exchanger: Synthesis, characterization and applications for the efficient removal of Pb^2+^ ion from aqueous medium. J. Ind. Eng. Chem..

[19] Naushad M., ALOthman Z.A., Javadian H. (2015). Removal of Pb(II) from aqueous solution using ethylene diamine tetra acetic acid-Zr(IV) iodate composite cation exchanger: Kinetics, isotherms and thermodynamic studies. J. Ind. Eng. Chem..

[20] Naushad M. (2014). Surfactant assisted nano-composite cation exchanger: Development, characterization and applications for the removal of toxic Pb^2+^ from aqueous medium. Chem. Eng. J..

[21] Ali I. (2012). New Generation Adsorbents for Water Treatment. Chem. Rev..

[22] Congeevaram S., Dhanarani S., Park J., Dexilin M., Thamaraiselvi K. (2007). Biosorption of chromium and nickel by heavy metal resistant fungal and bacterial isolates. J. Hazard. Mater..

[23] Sun F., Shao Z. (2007). Biosorption and bioaccumulation of lead by *Penicillium* sp. Psf-2 isolated from the deep sea sediment of the Pacific Ocean. Extremophiles.

[24] Vijayaraghavan K., Padmesh T.V.N., Palanivelu K., Velan M. (2006). Biosorption of nickel(II) ions onto *Sargassum wightii*: Application of two-parameter and three-parameter isotherm models. J. Hazard. Mater..

[25] Vijayaraghavan K., Yun Y.S. (2008). Bacterial biosorbents and biosorption. Biotechnol. Adv..

[26] Vieira R.H.S.F., Volesky B. (2000). Biosorption: A Solution to pollution. Int. Mycrobiol..

[27] Remacle J. (1990). The Cell Wall and Metal Binding. Biosorption of Heavy Metals.

[28] Prasenjit B., Sumathi S. (2005). Uptake of chromium by *Aspergillus foetidus*. J. Mater. Cycles Waste Manag..

[29] Bautista-Hernández D.A., Ramírez-Burgos L.I., Duran-Páramo E., Fernández-Linares L. (2012). Zinc and Lead Biosorption by *Delftia tsuruhatensis*: A Bacterial Strain Resistant to Metals Isolated from Mine Tailings. J. Water Resour. Protect..

[30] Maldonado J., de los Rios A., Esteve I., Ascaso C., Puyen Z.M., Brambilla C., Solé A. (2010). Sequestration and in vivo effect of lead on DE2009 microalga, using high-resolution microscopic techniques. J. Hazard. Mater..

[31] Shin M.-N., Shim J., You Y., Myung H., Bang K.S., Cho M., Kamala-Kannan S., Oh B.-T. (2012). Characterization of lead resistant endophytic *Bacillus* sp. MN3-4 and its potential for promoting lead accumulation in metal hyperaccumulator *Alnus firma*. J. Hazard. Mater..

[32] Bai J., Yang X., Du R., Chen Y., Wang S., Qiu R. (2014). Biosorption mechanisms involved in immobilization of soil Pb by *Bacillus subtilis* DBM in a multi-metal-contaminated soil. J. Environ. Sci..

[33] Naik M.M., Dubey S.K. (2013). Lead resistant bacteria: Lead resistance mechanisms, their applications in lead bioremediation and biomonitoring. Ecotoxicol. Environ. Saf..

[34] Chen Z., Pan X., Chen H., Guan X., Lin Z. (2016). Biomineralization of Pb(II) into Pb-hydroxyapatite induced by Bacillus cereus 12-2 isolated from Lead-Zinc mine tailings. J. Hazard. Mater..

[35] Ullah A., Heng S., Munis M.F.H., Fahad S., Yang X. (2015). Phytoremediation of heavy metals assisted by plant growth promoting (PGP) bacteria: A review. Environ. Exp. Bot..

[36] Filella M., Belzile N., Lett M.-C. (2007). Antimony in the environment: A review focused on natural waters. III. Microbiota relevant interactions. Earth Sci. Rev..

[37] Li D., Xu X., Yu H., Han X. (2017). Characterization of Pb^2+^ biosorption by psychrotrophic strain *Pseudomonas* sp. I3 isolated from permafrost soil of Mohe wetland in Northeast China. J. Environ. Manag..

[38] Çolak F., Atar N., Yazıcıoğlu D., Olgun A. (2011). Biosorption of lead from aqueous solutions by Bacillus strains possessing heavy-metal resistance. Chem. Eng. J..

[39] Matyara F., Kayab A., Dinçerb S. (2008). Antibacterial agents and heavy metal resistance in gram-negative bacteria isolated from seawater, shrimp and sediment in iskenderun bay, turkey. Sci. Total Environ..

[40] Beveridge T.J. (1989). Role of Cellular Design in Bacterial Metal Accumulation and Mineralization. Ann. Rev. Microbiol..

[41] Da Costa A.C.A., Ebinghaus R. (1999). Chemical Interactions between Mercurial Species and Surface Biomolecules from Structural Components of Some Biological Systems. Mercury Contaminated Sites: Characterization, Risk Assessment and Remediation.

[42] Dursun A.Y., Uslu G., Cuci Y., Aksu Z. (2003). Bioaccumulation of copper(II), lead(II) and chromium(VI) by growing *Aspergillus niger*. Process Biochem..

[43] Hemambika B., Rani M.J., Kannan V.R. (2011). Biosorption of heavy metals by immobilized and dead fungal cells: A comparative assessmen. J. Ecol. Nat. Environ..

[44] Aksu Z. (2005). Application of biosorption for the removal of organic pollutants: A review. Process Biochem..

[45] Turpeinen R., Kairesalo T., Häggblom M.M. (2004). Microbial community structure and activity in arsenic-, chromium- and copper-contaminated soils. FEMS Microbiol. Ecol..

[46] MIDI (1995). MIDI Sherlock Microbial Identification System Operating Manual.

[47] Das N. (2010). Recovery of precious metals through biosorption—A review. Hydrometallurgy.

[48] Langmuir I. (1918). The adsorption of gases on plane surfaces of glass, mica and platinum. J. Chem. Phys..

[49] Freundlich H. (1906). Uber Die Adsorption in Lasungen. J. Phys. Chem..

[50] Isik M. (2008). Biosorption of Ni(II) from aqueous solutions by living and non-living ureolytic mixed culture. Colloids Surf. B Biointerfaces.

[51] Günay A., Arslankaya E., Tosun İ. (2007). Lead removal from aqueous solution by natural and pretreated clinoptilolite: Adsorption equilibrium and kinetics. J. Hazard. Mater..

[52] Dubinin M.M., Zaverina E.D., Radushkevich L.V. (1947). Sorption and Structure of Active Carbons I. Adsorption of Organic Vapors. Zhurnal Fizicheskoi Khimii.

[53] Lagergren S.Y. (1898). ur theorie der sogenannten adsorption geloster stoffe, Kungliga Svenska Vetenskapsakademiens. Handlingar.

[54] Ho Y.S., McKay G. (1999). Pseudo-second order model for sorption processes. Process Biochem..

[55] Lodeiro P., Barriada J.L., Herrero R., De Vicente M.S. (2006). The marine macroalga *Cystoseira baccata* as biosorbent for cadmium(II) and lead(II) removal: Kinetic and equilibrium studies. Environ. Pollut..

[56] Woodburn J.M.Q.Y.G. (1999). Biosorption of cadmium from aqueous solutions by pretreated biomass of marine alga *Durvillaea potatorum*. Water Res..

[57] Karthikeyan S., Balasubramanian R., Iyer C.S.P. (2007). Evaluation of the marine algae *Ulva fasciata* and *Sargassum* sp. for the biosorption of Cu(II) from aqueous solutions. Bioresour. Technol..

[58] Panda G.C., Das S.K., Chatterjee S., Maity P.B., Bandopadhyay T.S., Guha A.K. (2006). Adsorption of cadmium on husk of *Lathyrus sativus*: Physico-chemical study. Colloids Surf. B Biointerfaces.

[59] Anayurt R.A., Sari A., Tuzen M. (2009). Equilibrium, thermodynamic and kinetic studies on biosorption of Pb(II) and Cd(II) from aqueous solution by macrofungus (*Lactarius scrobiculatus*) biomass. Chem. Eng. J..

[60] Uluozlu O.D., Sari A., Tuzen M., Soylak M. (2008). Biosorption of Pb(II) and Cr(III) from aqueous solution by lichen (*Parmelina tiliaceae*) biomass. Bioresour. Technol..

[61] Mckay Y.H.G. (2000). The kinetics of sorption of divalent metal ions onto sphagnum moss peat. Water Res..

[62] Veglio F., Beolchini F. (1997). Removal of metals by biosorption: A review. Hydrometallurgy.

[63] Martins B.L., Cruz C.C., Luna A.S., Henriques C.A. (2006). Sorption and desorption of Pb^2+^ ions by dead *Sargassum* sp. biomass. Biochem. Eng. J..

[64] Wang J., Chen C. (2006). Biosorption of heavy metals by *Saccharomyces cerevisiae*: A review. Biotechnol. Adv..

[65] David Kratochvil B.V. (1998). Advances in the biosorption of heavy metals. Trends Biotechnol..

[66] Parvathi K., Nagendran R., Kumar R.N. (2007). Lead biosorption onto waste beer yeast by-product, a means to decontaminate effluent generated from battery manufacturing industry. Electron. J. Biotechnol..

[67] Zhang D., Pan X., Zhao L., Mu G. (2011). Biosorption of Antimony (Sb) by the Cyanobacterium *Synechocystis* sp.. Pol. J. Environ. Stud..

[68] Pan J., Ge X., Liu R., Tang H. (2006). Characteristic features of *Bacillus cereus* cell surfaces with biosorption of Pb(II) ions by AFM and FT-IR. Colloids Surf. B Biointerfaces.

[69] Wu F., Sun F., Wu S., Yan Y., Xing B. (2012). Removal of antimony(III) from aqueous solution by freshwater cyanobacteria Microcystis biomass. Chem. Eng. J..

[70] Tunali S., Çabuk A., Akar T. (2006). Removal of lead and copper ions from aqueous solutions by bacterial strain isolated from soil. Chem. Eng. J..

[71] Pan J.-H., Liu R.-X., Tang H.-X. (2007). Surface reaction of Bacillus cereus biomass and its biosorption for lead and copper ions. J. Environ. Sci..

[72] Filella M., Belzile N., Chen Y.-W. (2002). Antimony in the environment: A review focused on natural waters: I. occurrence. Earth Sci. Rev..

